# Influence of Temperature on the Optical Properties of Ternary Organic Thin Films for Photovoltaics

**DOI:** 10.3390/ma18143319

**Published:** 2025-07-15

**Authors:** Gabriela Lewinska, Jerzy Sanetra, Konstanty W. Marszalek, Alexander Quandt, Bouchta Sahraoui

**Affiliations:** 1 Institute of Electronics, AGH University of Krakow, 30 Mickiewicza Ave, 30-059 Krakow, Poland; marszale@agh.edu.pl; 2 Advanced Diagnostic Equipment sp. zoo, Włodzimierza Tetmajera 79, 31-352 Krakow, Poland; jsanetra@agh.edu.pl; 3 Univ Angers, LPhiA, SFR MATRIX, F-49000 Angers, France; alex.quandt@wits.ac.za (A.Q.); bouchta.sahraoui@univ-angers.fr (B.S.); 4 School of Physics, University of the Witwatersrand, Private Bag 3, Johannesburg 2050, South Africa

**Keywords:** spectroscopic ellipsometry, organic materials, thin films, third harmonic generation

## Abstract

This study investigates the influence of temperature on the linear and nonlinear optical properties of ternary organic thin films for solar cell applications. Three-component organic thin films (poly({4,8-bis[(2-ethylhexyl)oxy]benzo [1,2-b:4,5-b′]dithiophene-2,6-diyl}{3-fluoro-2-[(2-ethylhexyl)carbonyl] thieno[3,4-b]thiophenediyl}) and (poly([2,6′-4,8-di(5-ethylhexylthienyl)benzo[1,2-b;3,3-b]dithiophene]{3-fluoro-2[(2-ethylhexyl)carbonyl]thieno[3,4-b]thiophenediyl}), marked PTB7 and PTB7th- donors, PCBM, phenyl-C61-butyric acid methyl ester acceptor, and Y5: 2,2′-((2Z,2′Z)-((12,13-bis(2-ethylhexyl)-3,9-diundecyl-12,13-dihydro[1,2,5]thiadiazolo[3,4e]thieno[2′,3′:4′,5′] thieno[2′,3′:4,5]pyrrolo[3,2-g] thieno[2′,3′:4,5]thieno[3,2-b]indole-2,10-diyl)bis(methanylylidene))bis(3-oxo-2,3-dihydro1H-indene-2,1-diylidene))dimalononitrile) and Y6 non-fullerene acceptors: (2,2′-((2Z,2′Z)-((12,13-bis(2-ethylhexyl)-3,9-diundecyl-12,13- dihydro-[1,2,5]thiadiazolo[3,4- e] thieno [2,″3″:4′,5′]thieno [2′,3′:4,5]), non-fullerene acceptors, were analyzed using spectroscopic ellipsometry and third-harmonic generation techniques across a temperature range of 30 °C to 120 °C. The absorption spectra of the ternary layers remained largely stable with temperature, but ellipsometry revealed temperature-dependent changes in layer thickness (a few percent increase during heating) and variations in refractive index and extinction coefficients, suggesting modest structural alterations. Analysis using a gradient model indicated that film composition varies with thickness. Third-harmonic generation measurements showed a decrease in χ^(3)^ after annealing, with the most significant change observed in the PTB7th:Y5:PCBM layer.

## 1. Introduction

Organic semiconductors are breakthrough materials for lightweight portable flexible electronics. The use of organic electronics is a growing field of research and application. Luminescent diodes [1,2,3], photovoltaic cells [4,5,6], organic transistors [7,8,9], and memristors [10,11,12], among others, are being developed. Since Tang [13] new materials and technologies have been developed for organic solar cells (OSCs). The incorporation of processing additives is one of the most prevalent techniques for optimizing bulk heterojunction solar cells; therefore, one of the developing trends in organic photovoltaics is ternary organic solar cells (TOSCs) [14,15]. An additional donor or acceptor is added to the traditional active layer in a bulk heterojunction solar cell to provide better absorbance and improved charge transport [16]. As a result, better and better TOSC efficiencies of 19–20% are being achieved [17,18,19,20]. Ternary organic solar cells [21,22] still have the potential to improve performance over binary arrangements, as has been highlighted by a recent study. A third component that can optimize electronic characteristics, tune morphology, and increase spectrum absorption is incorporated into TOSCs [23]. Power conversion efficiency has surpassed 17% as a result of this approach [24]. One noteworthy accomplishment is the use of a novel non-fullerene acceptor that improves molecular packing and lowers voltage loss by forming a homogeneous mixed phase with existing components. According to the active layer materials, TOSCs may be divided into four types: all-polymer, all-small-molecule, polymer/small molecule/small molecule, and polymer/polymer/small molecule [25]. Advancements in efficiency are bringing these solar cells closer to realizing their full potential, as evidenced by recent demonstrations [26,27]. As one of the most important thermal management components in organic electronic systems, organic material-based thermal switches are gaining a lot of attention [28]. The development of thermoelectric devices has prompted a lot of interest in small organic semiconductors [29].

However, all these devices operate in an external environment, so it is necessary to determine the influence of environmental factors such as temperature, open atmosphere, and (intense) radiation on their performance. In the case of degradation under the influence of some of these external factors, one could use encapsulation techniques to prevent them [30], but temperature changes are impossible to exclude. Therefore, it is necessary to study how temperature affects the properties of the most essential part of a solar cell—the active film. When the temperature of a silicon solar cell increases, its saturation current density increases exponentially, because the band gap of silicon decreases with increasing temperature, and additional low energetic photons can be absorbed. On the other hand, the open circuit voltage decreases linearly, and this effect outweighs the gain in saturation current density. The net effect is a reduction in solar cell efficiency with increasing temperature [31]. In the case of thin organic layers and therefore organic solar cells, these properties are even more complicated, and reported results are ambiguous. In such a situation, it certainly makes sense to choose only one aspect and carefully study its influence on the properties of the active layers in an organic solar cell.

The present study will focus on the optical properties of ternary layers for organic solar cells at the recommended maximum temperatures and beyond, which refer to situations that are frequently encountered during the practical operation of such devices. For example, studies of differential scanning calorimetry (DSC) are often carried out because of the volume effect in thin films, the thermal properties of which can vary. Temperature annealing is frequently used to improve the quality of the (two- or three-component) active layer. To this end, the active layer in organic cells was heated to 390 K (177 °C) during deposition, as described by Zheng et al. [32], to improve the power conversion efficiency. Likewise, the active layer was annealed at 120 °C for 10 min by Green et al. [33], at 60–80 °C by Thao [34], at 150 °C by Miao et al. [35], and at 160 °C by Shaban et al. [36] (efficiency values in cells with thermal treatment are collected in Supplementary Materials in Table S5). The operation temperature varied according to various reports between 60 °C [37], 70 °C [38], and 80 °C [39], which is within the scope of the International Standard IEC 61215 [38]. However, the standard indicates that there are instances where users may want to consider testing under test conditions corresponding to higher temperatures than those described, which was also the main motivation for increasing the temperature range in our study.

Concerning the active layers in bulk heterojunction solar cells, selected chemical compounds have been used as components of these active layers, and often in combination with other materials (e.g., fullerene acceptors). Previous studies presented in the literature describe the resulting devices and their parameters. In this work, we focus on the optical aspect of the photovoltaic process. In recent studies, we have developed research on binary active layers [40]. However, the following investigations are driven by the increasing interest in TOSC. In this work, we focused on ternary combinations with two thiophene-based donors, (PTB7 and PTB7th, [41,42]: poly({4,8-bis[(2-ethylhexyl)oxy]benzo[1,2-b:4,5-b′]dithiophene-2,6-diyl}{3-fluoro-2-[(2-ethylhexyl)carbonyl] thieno[3,4-b]thiophenediyl}) and (poly([2,6′-4,8-di(5-ethylhexylthienyl)benzo[1,2-b;3,3-b]dithiophene]{3-fluoro-2[(2-ethylhexyl)carbonyl]thieno[3,4-b]thiophenediyl}), respectively), an acceptor fullerene (PCBM, phenyl-C61-butyric acid methyl ester) and two non-fullerene acceptors, designated Y5: 2,2′-((2Z,2′Z)-((12,13-bis(2-ethylhexyl)-3,9-diundecyl-12,13-dihydro[1,2,5]thiadiazolo[3,4e]thieno[2′,3′:4′,5′] thieno[2′,3′:4,5]pyrrolo[3,2-g] thieno[2′,3′:4,5]thieno[3,2-b]indole-2,10-diyl)bis(methanylylidene))bis(3-oxo-2,3-dihydro1H-indene-2,1-diylidene))dimalononitrile) and Y6: (2,2′-((2Z,2′Z)-((12,13-bis(2-ethylhexyl)-3,9-diundecyl-12,13- dihydro-[1,2,5]thiadiazolo[3,4- e] thieno [2″,3″:4′,5′]thieno [2′,3′:4,5]). The combinations of ternary mixtures like PTB7:Y5:PCBM, PTB7th:Y5:PCBM, PTB7th:Y6:PCBM, and PTB7th:Y6:PCBM are particularly interesting due to their wide applicability in ternary organic cells [43,44].

Note that the PTB7 semiconducting copolymer comprising thieno[3,4-b]thiophene and benzodithiophene alternating repeat units provides a historic record of solar energy conversion efficiency (7.4%) in polymer/fullerene bulk heterojunction solar cells. Thermal changes for PTB7 and PTB7th compounds have been described for temperatures above 300 °C via thermogravimetry and DSC, as well as temperature-induced changes for PCBMs [45]. For PCBM, Hajduk et al. reported changes in ellipsometric angles at 103 °C [46]. There were no endothermic or exothermic peaks recorded for Y6 in the selected temperature range, indicating good crystallinity and stability below 200 °C, according to Mahdy et al. [47].

In this study, we prepared three-component mixtures that correspond to the following configurations: PTB7:Y5:PCBM, PTB7:Y6:PCBM, PTB7th: Y5:PCBM, and PTB7th: Y6:PCBM. The sizes of PTB7th and PTB7 can vary depending on the polymer chain length, and polymer chains can extend from a few to several nanometers in length. Owing to its fullerene core, PCBM has a diameter of approximately 1 nm [48]. The sizes of Y5 and Y6 can be estimated to be a few nanometers, based on the size of the benzene ring.

For each of these ternary active layer materials, we focused on their optical properties under varying temperatures, which were measured via spectroscopic absorption spectroscopy [49,50], spectroscopic ellipsometry [51], and third harmonic generation techniques [52,53,54].

## 2. Materials and Methods

**Figure 1:**
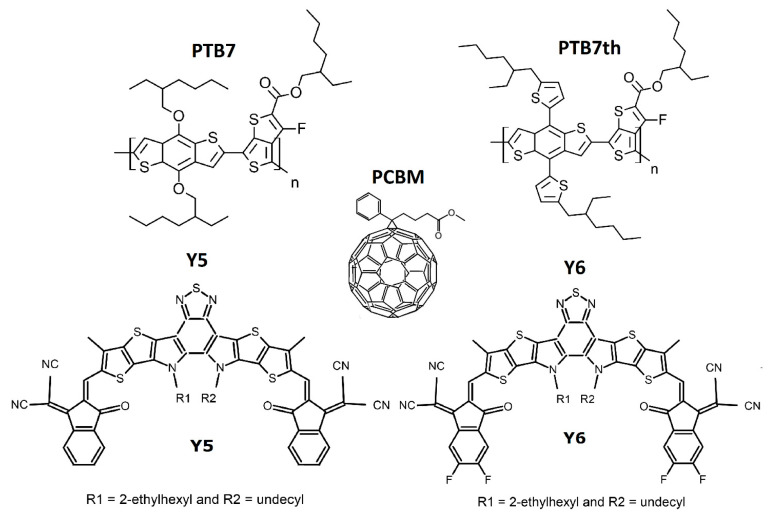
The chemical formulas of the compounds under investigation PTB7, PTB7th, PCBM, Y5, Y6.

The materials and reagents used were purchased from Merck KGaA (Darmstadt, Germany). The chemical formulas of the compounds under investigation are shown in Figure 1. The compounds were dissolved in chloroform (spectroscopic grade), mixed as 1:1:1 solutions by weight, and applied to the substrate via the spin coating method. A spin coater model VTC-100 was used (MTI Corporation, Richmond, VA, USA). For spectroscopic studies, the preferred substrate was quartz; for ellipsometry, the preferred substrate was silicon; and for THG (third harmonic generation) studies, the preferred substrate was glass.

UV‒Vis spectroscopy was performed with an Avantes Sensline Ava-Spec ULS-RSTEC spectrophotometer (Avantes, Appelsdorn, The Netherlands). The measurement range for Uv-Vis spectroscopy was from 250 nm to 1000 nm. Spectroscopic ellipsometry measurements were performed using an M-2000 spectroscopic ellipsometer (J.A. Woollam Co., Lincoln, NE, USA). Data (collected ellipsometric angles) were made in a wider scope (192–1700 nm). Fits were made in the range of 340–1700 nm. The tests were performed at three angles (65°, 70°, 75°).

The Maker Fringes method has been used for third-harmonic generation [55,56]. The apparatus was a pulsed Nd: YAG laser Ekspla, PL2250 (©Ekspla, Vilnius, Lithuania) with a pulse duration of 30 ps, a wavelength of 1064 nm, and a frequency of 10 Hz.

UV‒Vis spectroscopy and ellipsometry studies were conducted in situ during the heating and cooling of the film. The temperature was stabilized for approximately one minute, and the measurement range was from 30 °C to 120 °C and from 120 °C back to 30 °C. THG measurements were carried out first for the samples before annealing, then for the samples heated to 120 °C, cooled to 30 °C, and then retested. The reference material for determining the THG was quartz.

Sample preparation was initiated by preparing solutions from the crystalline phase of each component at a concentration of 10 mg/mL. The solutions were dissolved in spectral chloroform. The target mixtures under consideration was then made at a proportion of 1:1:1. The substrates used were previously cleaned in an ultrasonic cleaner. The samples were deposited in the 23 °C temperature in argon atmosphere. The samples were store 24 h for removal of residual solvent.

## 3. Results and Discussion

### 3.1. UV–Vis Spectroscopy

**Figure 2:**
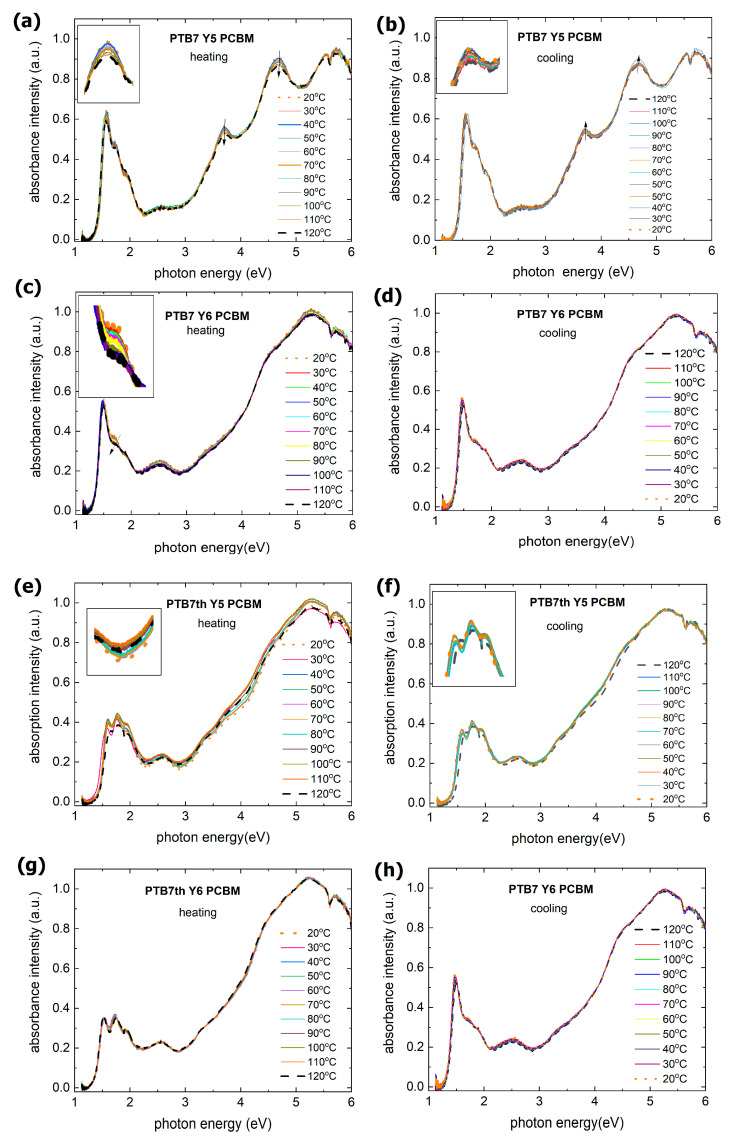
UV-VIS spectra vs. photon energy of ternary layers during heating (from 20 °C to 120 °C) and cooling (from 120 °C to 20 °C) of various thin films: (**a**,**b**) PTB7:Y5:PCBM, (**c**,**d**) PTB7:Y6:PCBM, (**e**,**f**) PTB7th:Y5:PCBM, and (**g**,**h**) PTB7th:Y6:PCBM.

Photon absorption and the creation of an electron-hole pair is the first phase of the photovoltaic process. Therefore, absorbance tests were carried out in the range of 350 nm to 1100 nm for temperatures ranging from 30 °C to 120 °C, after which the samples were subsequently cooled back to 30 °C. The absorption spectra of the various mixed layers are shown in Figure 2.

Ternary active layers have a broad absorption band covering a large part of the solar spectrum, with absorption maxima that depend on the chosen composition. One of these absorption maxima is typically around 500 nm (solar spectral maximum), and the other three maxima are located in the band from 600 nm to 850 nm. The PTB7:Y5:PCBM layer showed an additional maximum of 375 nm.

For each of the ternary layers, the absorption spectrum is temperature stable, showing no change in either peak position or intensity. The only slight deviations appear for the PTB7th:Y5:PCBM layer at 120 °C. There is a slight trend in PTB7:Y5:PCBM plots; as suggested we have added arrows. The changes are due to the broadening/smearing of atomic levels with the change in temperature.

### 3.2. Ellipsometry

Ellipsometry is a widely used, efficient technique for determining fundamental optical constants (refractive indices, extinction coefficients), dielectric constants (ε_1,_ ε_2_), and thin film thicknesses, and it provides information about the structural order of a given layer, such as its anisotropy. These characteristics must be extracted from the recorded ellipsometric spectra through modeling. After fitting a model suitable for the material under study, a range of information about thin film properties may be obtained from the measured values of the ellipsometry angles [57].

Ellipsometric measurements were performed for determining fundamental optical constants (refractive indices, extinction coefficients) and thin film thicknesses.

For the present study, a model based on oscillators (GenOsc^TM^), which is consistent with the Kramers–Kronig relation, was used to fit the layers under investigation [58]. Note that various types of oscillator functions are typically used to define the unknown complex dielectric function generally defined as

ε = ε_1_ + iε_2_ (1)

where ε_1,_ ε_2_ dielectric constants, connected with complex refractive index N

(2) N ≡n−ik

as $N^{2}\equiv \varepsilon$, n is the refractive index, and k is the extinction coefficient.

To be more specific, $\varepsilon \left(E\right)$ in Equation (1) represents the dielectric function as a function of energy

(3) $$\varepsilon \left(E\right)=A_{n}\left(\Gamma \left(\frac{E+E_{n}}{\sigma_{n}}\right)+i\left[e^{-{\left(\frac{E-E_{n}}{\sigma_{n}}\right)}^{2}}+e^{-{\left(\frac{E+E_{n}}{\sigma_{n}}\right)}^{2}}\right]\right)$$

where An is the amplitude of the chosen oscillator model, Γ is a convergent series that produces the Kramer’s Kroning relation, En is the center energy of the chosen Gaussian oscillator model, and $\sigma_{n}$ is described by Equation (4) (where Br represents the broadening of the oscillator). Selected values of oscillator parameters are included in Supplementary Materials (Tables S1–S4).

(4) $$\sigma_{n}=\frac{Br_{n}}{2\sqrt{ln2}}$$

In the investigated system of heated samples, the optical properties of the system were modeled as a gradient (i.e., a set of layers of different optical characteristics), and the active layer is understood to be a mixed homogeneous blend. This model must be contrasted to the fact that organic polymeric-fullerene layers have been shown to have a gradient refractive index and extinction coefficient [59]. Depending on the composition, the optical characteristics and their inhomogeneity can vary to a lesser or larger degree.

**Figure 3:**
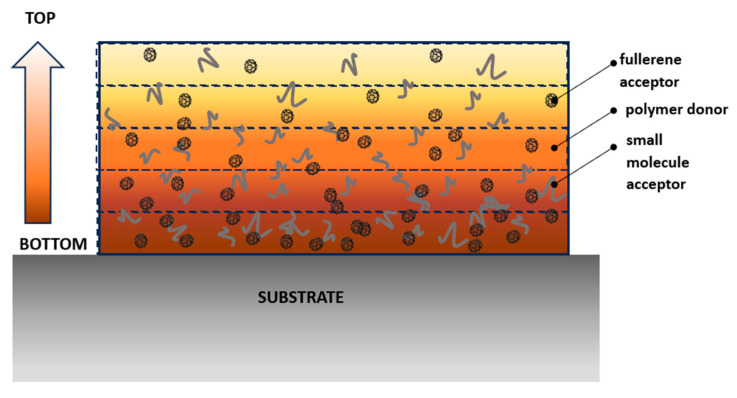
Scheme of the gradient thin film on substrate. Molecule dimensions are conventional and do not represent actual size proportions.

**Figure 4:**
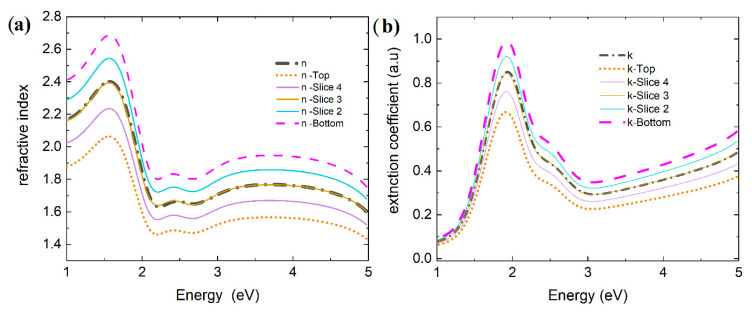
Fitting a gradient model of the refractive index (**a**) and extinction coefficient (**b**) vs. photon energy for PTB7th:Y5:PCBM.

The scheme of the gradient thin film on substrate was presented in Figure 3. The layer is divided into five slides, each of which is characterized by its specific index of refraction and extinction coefficient (n and k-slide), and the average value of n and k (black dash-dot line). The graded simple model was used for five stripes. A schematic of the layer model fitting concept is shown in Figure 4. The optical coefficients of the top layer (n and k-Top) and the bottom layer (n and k-Bottom) are also shown in these figures.

**Figure 5:**
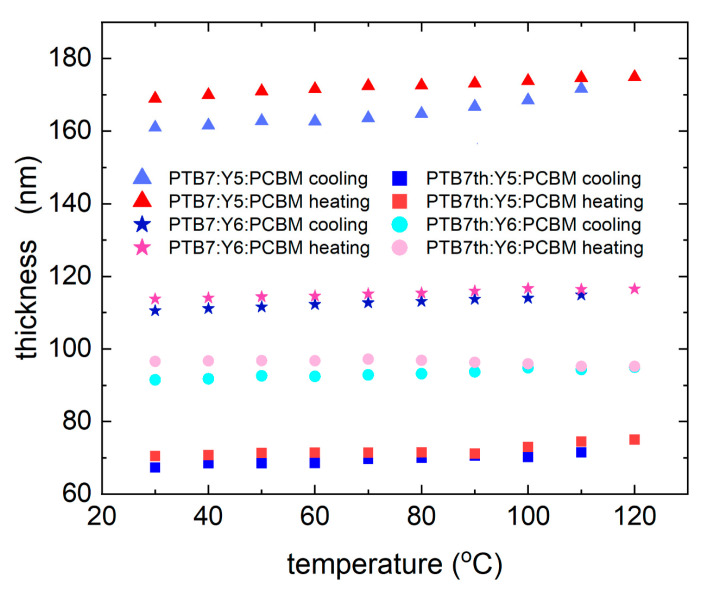
Layer thickness changes for the investigated thin films during heating (from 30 °C to 120 °C) and cooling (from 120 °C to 30 °C).

As a result of the fit to the ellipsometry data, we could also quantify changes in the layer thickness as a function of temperature (Figure 5). Each of the three-component layers increases in thickness during heating and then slightly decreases in thickness during cooling. The PTB7:Y5:PCBM layer demonstrated an increase of 6 nm (3% relative to the layer thickness) during heating and a decrease of 5 nm (6%) during cooling. For the PTB7:Y6:PCBM layer, an increase in thickness was found with heating by 7 nm (6% of the layer thickness) and 3 nm (2% of the layer thickness). The thicknesses of the PTB7th Y5:PCBM and PTB7th:Y6:PCBM layers changed with heating by 11 nm (10% of the layer thickness) and 4 nm (4% of the layer thickness) and with cooling by 5 nm (6%) and 2 nm (2%), respectively. Thus, the thickness changes with the temperature by approximately a few percent for all the layers. These changes are quite smooth, and no rapid transformations were observed.

**Figure 6:**
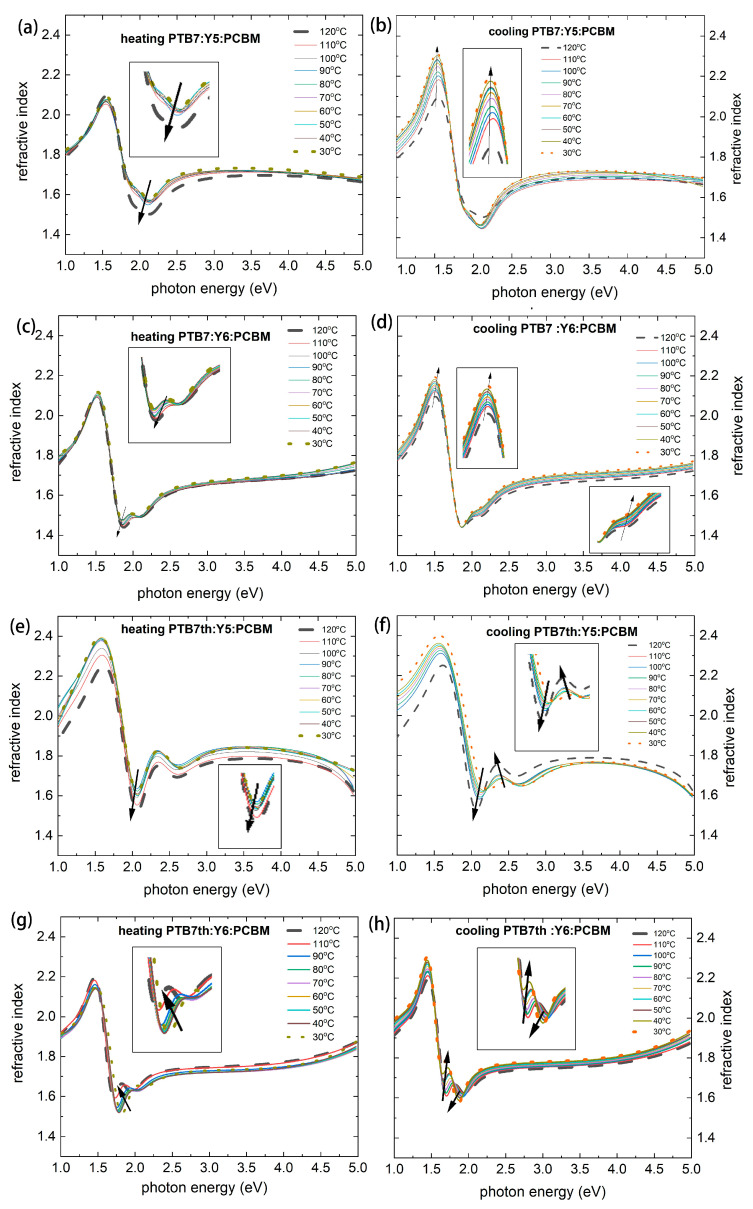
The refractive indices n as a function of photon energy during heating (from 30 °C to 120 °C) and cooling (from 120 °C to 30 °C) of thin films (**a**,**b**), PTB7:Y5:PCBM, (**c**,**d**) PTB7:Y6:PCBM, (**e**,**f**) PTB7th:Y5:PCBM, and (**g**,**h**) PTB7th:Y6:PCBM.

**Figure 7:**
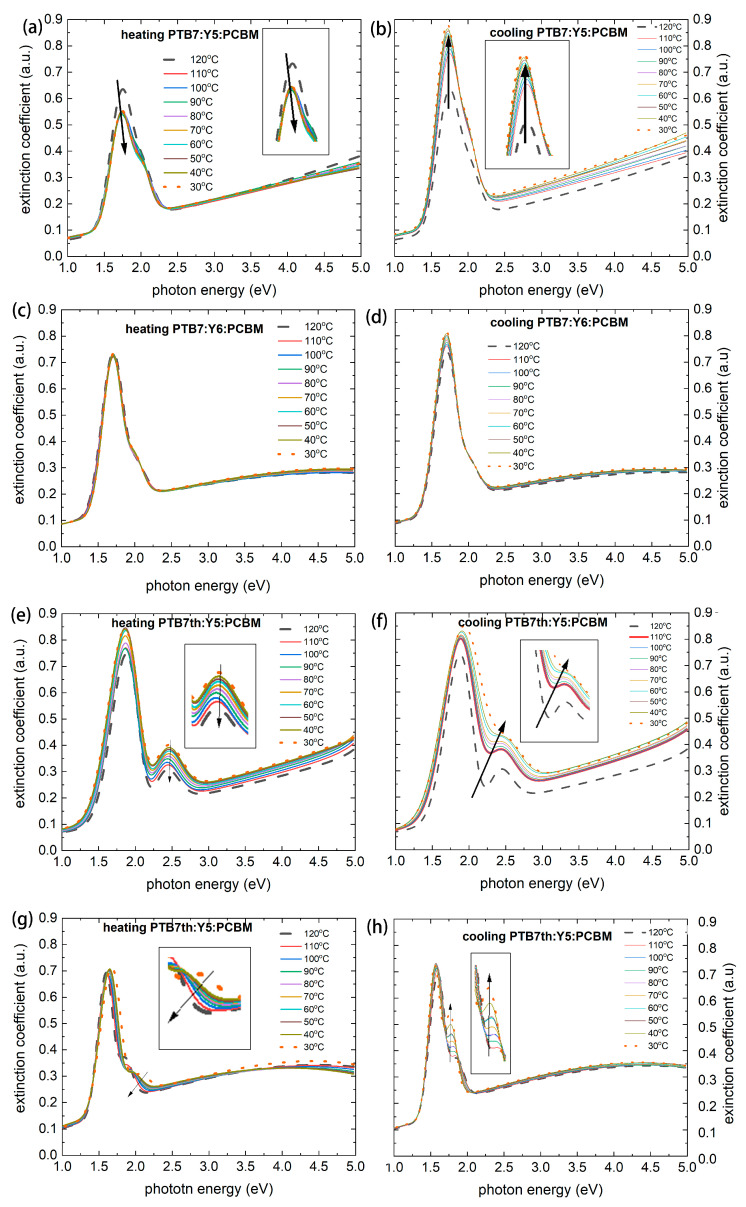
The extinction coefficients k during heating vs. photon energy (from 30 °C to 120 °C) and cooling (from 120 °C to 30 °C) of thin films PTB7:Y5:PCBM (**a**,**b**), PTB7:Y6:PCBM (**c**,**d**), PTB7th:Y5:PCBM (**e**,**f**), and PTB7th:Y6:PCBM (**g**,**h**).

The thermal changes in the refractive and extinction coefficients over the considered range are shown in Figure 6 (refractive indices) and Figure 7 (extinction coefficients).

In layers PTB7:Y5:PCBM and PTB7th:Y5:PCBM (Figure 6a,c,e,f and Figure 7a,c,e,f), a decrease in the refractive index can be observed at a temperature of 120 °C, and the refractive index increases again during cooling. Changes in the extinction coefficient are also observed. In the PTB7th: Y6:PCBM film, small changes appear (an increase in the case of cooling for the extinction coefficient) within the range of approximately 1.6 eV to 1.8 eV. The highest stability of these fundamental optical properties (i.e., small variations in the extinction coefficient and refractive index) was observed for PTB7th: Y6: PCBM; even if an increase in the refractive index was also detected in this case.

A major change (shift) in the refractive index and extinction coefficient values is observed for the PTB7th: Y5:PCBM value at 110 °C and 120 °C. This confirms the changes already observed in the absorption spectrum. The peak shifts and intensity changes may indicate that crystallization or phase segregation occurred due to the thermal treatment.

**Figure 8:**
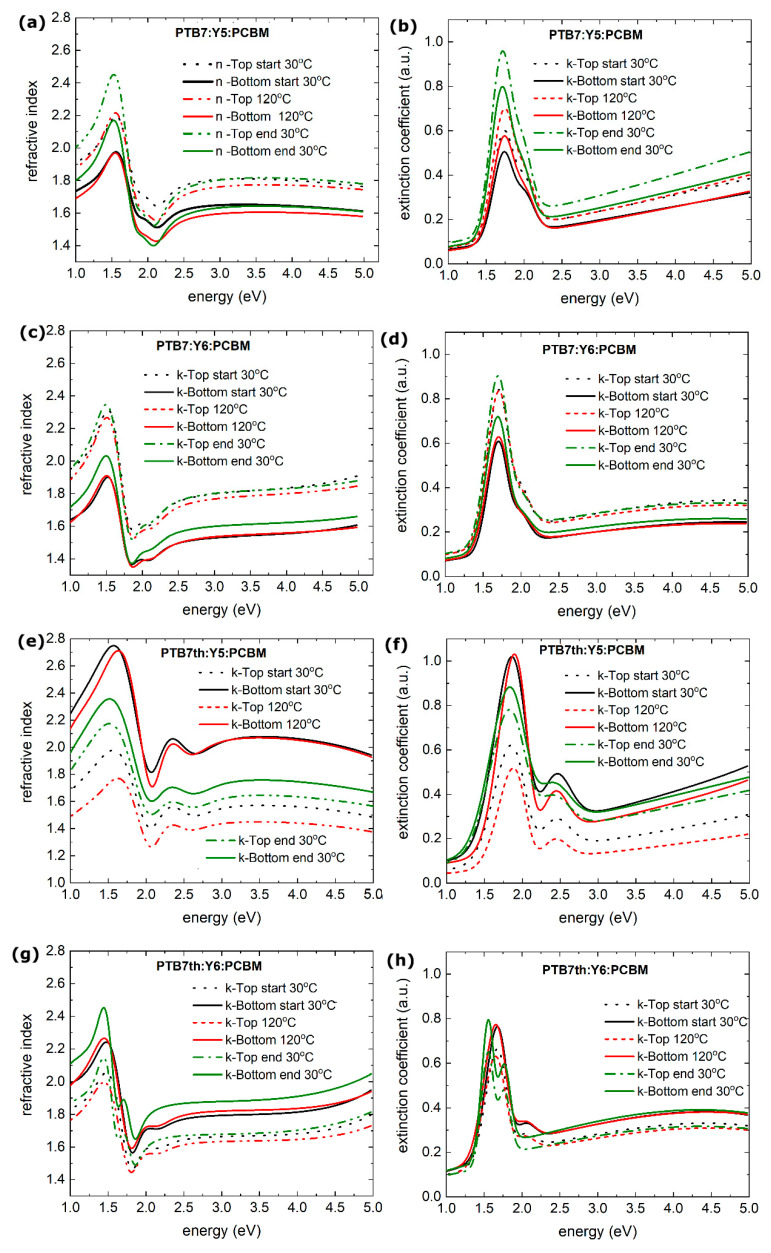
Gradient refractive indices vs. photon energy of thin film (**a**) PTB7:Y5:PCBM, (**c**) PTB7:Y6:PCBM, (**e**) PTB7th:Y5:PCBM, and (**g**) PTB7th:Y6:PCBM and gradient extinction coefficients of thin films (**b**) PTB7:Y5:PCBM, (**d**) PTB7:Y6:PCBM, (**f**) PTB7th: Y5:PCBM, and (**h**) PTB7th:Y6:PCBM for the beginning of the heating process (starting at 30 °C), at a temperature of 120 °C, and the cooling process (ending at 30 °C).

By extending our analysis to a gradient model, we obtain better insight into the variations in the optical properties for the layers during temperature changes (Figure 8).

The changes in the refractive indices between the upper parts of the PTB7:Y5:PCBM layer and the lower part of the layer changed from 0.2 during heating to 0.3 during cooling, and the extinction coefficient decreased. The inhomogeneity has changed by only a few percent (from 27% at 30 °C through 30% at 120 °C to 25% after the entire cycle at 30 °C).

The PTB7:Y6:PCBM layer shows inhomogeneity changes from 50% at 30 °C through 21% to 36% after the heating/cooling process. The difference between the upper and lower refractive indices is 0.3. The difference between the refractive indices of the top layer and the bottom layer is approximately 0.3, and the difference in the extinction coefficient is about 0.1.

The difference between top and bottom value of refractive index for the PTB7th:Y5:PCBM layer is about 0.8 for the entire range at 30 °C. After heating, it increases to 1.0 at 120 °C, and when the sample is cooled again at 30 °C, the difference changes to 0.2. For the extinction coefficient, the difference between the top and the bottom is approximately 0.4 at a temperature of 30 °C, and it increases to 1.0 at 120 °C, then decreases to 0.1 (after cooling to 30 °C). The inhomogeneity of the PTB7th:Y5:PCBM layer at 30 °C was 76%, increased to 87% upon heating, and then almost returned to its previous state, reaching a value of 74%.

In the case of the PTB7th:Y6:PCBM layers, the difference between the top and bottom layers of the thin film refractive index from 0.2 (30 °C) also increases slightly to 0.3 at 120 °C. When the layer is cooled from 120 °C to 30 °C, the refractive indices of the layers differ by approximately 0.4. The shape of the dispersion relation also changes, as could already be observed in the n and k dispersion spectra during heating and cooling. The inhomogeneity changes from 22% at 30 °C to 31% at 120 °C, and during cooling, it remains almost constant, reaching 32% at 30 °C.

For both layers based on the PTB7 donor, the “top” sublayers have a higher refractive index, as well as extinction coefficient, than the “bottom” layers do. For the layers containing the PTB7th donor, the opposite situation occurs: the “top” sublayers have a lower refractive index and extinction coefficient than the “bottom” layers do.

The differences between the top and bottom measurements suggest that the film composition varies with thickness. The changes in peak positions (for PTB7th:Y6:PCBM) can indicate a redistribution of components, with distinct domains forming. It is indeed known that changes in temperature significantly affect the structure and behavior of organic mixtures and colloidal systems in organic films, as previously reported [60,61,62].

Also, changes in refractive indices can be compensated for by changes in thickness, while keeping the absorption unchanged with varying temperatures. Thus, an improvement in the energy conversion efficiency of organic PCDTBT:PCBM solar cells has previously been observed without a significant change in optical absorption, which was induced by thermal annealing up to 150 °C [63].

For polymeric components, alterations in temperature could lead to changes in the polymer chain conformation. This process may induce slight phase segregation within the ternary blends, leading to a non-uniform distribution of the components. The resulting heterogeneity directly influences the refractive index and extinction coefficient profiles. It was reported by Oh-e et al. [64] that the heating of organic semiconductor films can result in randomization and constraint of molecular alignment, with anisotropy changes reported before the phase transition. Lungenschmied et al. [65] showed that temperature-induced transitions are associated with structural changes in the polymers and mixed morphology, with phase separation occurring at 120 °C.

### 3.3. Third Harmonic Generation

**Figure 9:**
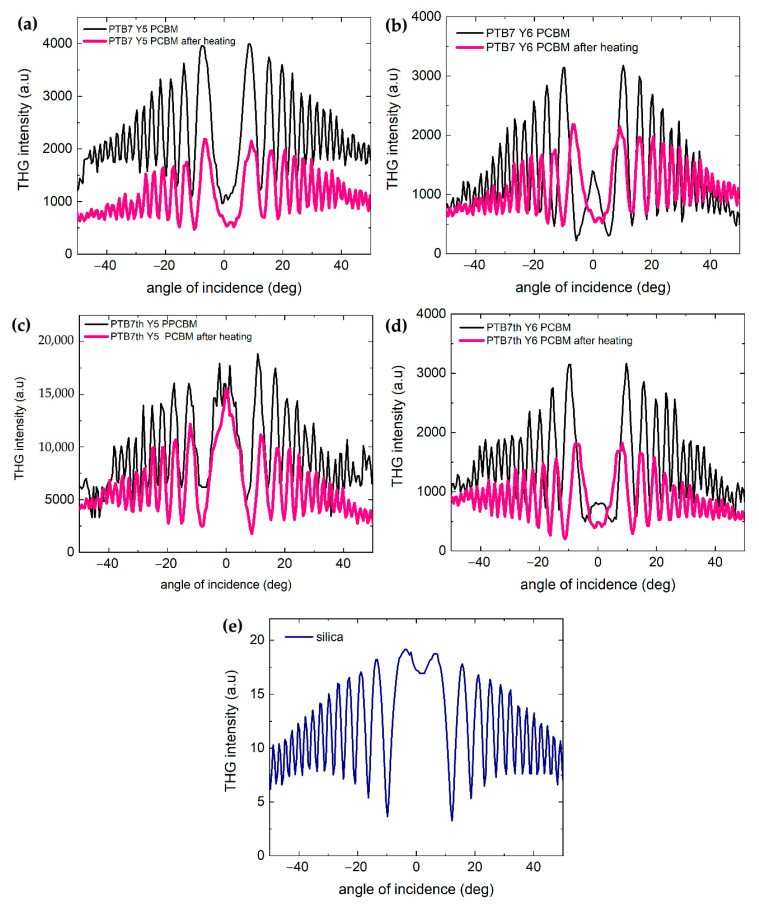
THG characterization of the investigated thin films (**a**) PTB7:Y5:PCBM, (**b**) PTB7:Y6:PCBM, (**c**) PTB7th:Y5:PCBM, (**d**) PTB7th:Y6:PCBM, and (**e**) reference material (quartz).

Another important aspect is the change of nonlinear optical properties with temperature. The third-harmonic generation (THG) phenomenon occurs when three photons interact to undergo a conversion process that produces photons with three times the optical frequency of the incoming photons. For the THG measurements, the unheated layer was studied first, followed by measurements after heating at 120 °C for 20 min and subsequent cooling. Third-harmonic measurements were carried out for all the materials considered before (Figure 9).

The most stable THG spectrum is obtained for PTB7:Y5:PCBM (with a thickness of 161 nm), which is a system with a stabilizing fullerene, a molecularly symmetric non-fullerene acceptor Y5, and a donor with two symmetric alkyl chains and one characteristic unsymmetric chain. The PTB7:Y6:PCBM (thickness of 128 nm) three-component layer shows a drastic change in the shape of the THG spectrum after annealing. For the layers based on donor PTB7, PTB7:Y5:PCBM, and PTB7:Y6:PCBM, satisfactory symmetry of the THG signal for both films was observed, confirming the smooth surface and solid quality of the films. Some symmetry of the THG signals at an incidence angle of 0° is observable for the PTB7th:Y5:PCBM (thickness of 255 nm) and PTB7th:Y6:PCBM (thickness of 142 nm) films.

The highest THG response was observed for the PTB7th:Y5:PCBM layer. In other cases, the THG intensity is approximately three thousand in arbitrary units. In the case of PTB7th:Y5:PCBM, there is a decrease in the spectrum after annealing (for maximum peaks of −8°/8°, there is a decrease after annealing from 4000 a.u. to 2200 a.u. The shape of the spectrum itself does not vary (the peaks remain in a similar position). This may be related to the greater charge transfer in the PTB7th:Y5:PCBM samples, which was observed by Yahya et al. [66].

Changes in the shape of the THG spectrum after annealing observed for PTB7:Y6:PCBM, PTB7th:Y5:PCBM, and PTB7th:Y6:PCBM may be connected with alterations in the molecular packing and structure within the film. Researchers previously reported that mixtures of Y-series acceptors create a glassy state, significantly reducing crystallization compared with single acceptor materials (confirmed through differential scanning calorimetry and fast scanning calorimetry) [60]. This suppressed crystallization is attributed to increased entropy upon mixing.

Model analysis of third-harmonic generation signals is used to determine the third-order susceptibility $\chi^{\left(3\right)}$. Throughout this study, the most suitable third-order nonlinear susceptibility $\chi^{\left(3\right)}$ model is described by the following formula:

(5) $$\chi^{\left(3\right)}=\chi_{Silica}^{\left(3\right)}\left(\frac{2}{\pi }\right)\left(\frac{L_{Silica}^{coh}}{d}\right)\sqrt{\frac{I^{3\omega }}{I_{Silica}^{3\omega }}}\left(\frac{\frac{\alpha \;d}{2}}{1-\exp^{-\frac{\alpha \;d}{2}}}\right)$$

Equation (5) is also known as the Kubodera and Kobayashi model [67], where

- d is the thickness of the sample,
- $I^{3\omega }$ is the THG maximum intensity value of the considered material,
- $I_{Silica}^{3\omega }$ is the THG maximum-intensity value of silica as the reference material,
- $\chi_{Silica}^{\left(3\right)}=2\cdot 10$^−22^ $\frac{m^{2}}{V^{2}}$ is the third-order susceptibility for silica,
- α is the linear absorption coefficient of the material at a wavelength of 355 nm,
- $L_{Silica}^{coh}=6.7$ μm is the coherence length of the silica glass.

The resulting nonlinear susceptibilies $\chi^{\left(3\right)}$ for the thin ternary films before and after annealing are presented in Table 1.

For all the layers after annealing, the values of χ^(3)^ have decreased. The smallest change was observed for PTB7:Y6:PCBM, and the largest change was observed for the PTB7th:Y5:PCBM, which was the most temperature-sensitive layer (a decrease in the χ^(3)^ value of almost 50%).

Such a reduction was also observed in the study by Hsu et al. [68], where effects of temperature on the photoinduced (THG) variation in an azo copolymer and an azo guest-host polymer were studied at two different temperatures. One possible explanation could be the changes in the intermolecular spacing and packing density within organic layers with varying temperatures. These structural changes can affect the degree of π‒π stacking, hydrogen bonding, and other intermolecular interactions, thus modifying the optical properties. Jung et al. [69] claimed that when the third component’s function is adequately matched with the binary host circumstances, it significantly improves the stability and efficiency of devices. The degree of inhomogeneity in the thin films, as revealed by the gradient model analysis, could affect the overall performance of the solar cell. Controlling the homogeneity of the active layer is crucial for achieving high device efficiency. According to Landerer et al. [70], the molecular and domain conformation of the ternary PTB7-Th:PDTP–DFBT:PC61BM solar cells conceals the source of their heat stability. The small molecule acceptors (not Y-type) proved to have a low tendency to form distinct phases because of their similar surface tensions [20], which may be connected with changes in film gradient and related to the reduction in the inhomogeneity of the considered layers during temperature changes.

## 4. Conclusions

A deeper understanding of changes in the fundamental optical properties of the active layer with varying temperatures provides us with interesting and important new information to develop more efficient types of organic solar cells. Thermal variations in the absorption, refractive index, and χ^(3)^ can affect the light-harvesting capability, charge carrier production, and transport in the active layer of a solar cell. Furthermore, thermal fluctuations are part of the manufacturing process of photovoltaic organic cells, as well as a common occurrence during their operation as a power production device, due to ambient temperature and weather variations.

The reported relative temperature stability of the absorption spectra indicate that the fundamental chemical structure of the ternary layers remains mostly intact within the studied temperature range (30–120 °C). However, the observed variations in the layer thickness, refractive index, and extinction coefficient suggest modest structural alterations, which depend on the ternary layer composition and temperature. These changes were attributed to possible structural rearrangements in the films.

We also observed that the THG varied significantly based on the layer composition and temperature, and we noticed a decrease in $\chi^{\left(3\right)}$ due to the annealing of the layer. These findings reveal the intricate relationship between the material composition, temperature, and optical characteristics of the ternary organic thin films.

By quantifying thermal variations in the optical properties of their active layers, one can properly distinguish these effects from other processes, such as the thermalization of charge carriers and heat transport within the solar cell, and thus arrive at a more detailed understanding of these complex phenomena.

## Figures and Tables

**Table 1:** Third-order nonlinear susceptibility of the investigated thin films before and after heating.

Sample	α (10^3^ cm^−1^) 355 nm	*χ*^(3)^(10^−22^$\frac{m^{2}}{V^{2}}$) Before Heating	*χ*^(3)^(10^−22^$\frac{m^{2}}{V^{2}}$) After Heating
PTB7:Y5: PCBM	42.64	272 ± 1.71	201± 1.25
PTB7:Y6: PCBM	53.84	246 ± 0.209	241 ± 2.62
PTB7th:Y5: PCBM	30.47	352 ± 0.116	181 ± 0.863
PTB7th:Y6: PCBM	79.34	442 ± 0.323	334 ± 0.244

## Data Availability

The raw data supporting the conclusions of this article will be made available by the authors on request.

## Supplementary Materials

The following supporting information can be downloaded at https://www.mdpi.com/article/10.3390/ma18143319/s1, Table S1. Gaussian oscillator parameters values for PTB7 Y5 PCBM. Table S2. Gaussian oscillator parameters values for PTB7 Y6 PCBM. Table S3. Gaussian oscillator parameters values for PTB7th Y5 PCBM. Table S4. Gaussian oscillator parameters values for PTB7th Y6 PCBM. Table S5. Efficiency values in cells with thermal treatment. Figure S1. Ellipsometry angles with the fitted model for the considered thin films during heating for 40 °C.
