# Supplementary material for: Influence of Temperature on the Optical Properties of Ternary Organic Thin Films for Photovoltaics
Source: Materials (Basel). 2025 Jul 15;18(14):3319. doi: 10.3390/ma18143319 (PMC12300046; doi:10.3390/ma18143319)
Supplement: Supplementary file 1 [file materials-18-03319-s001.zip › materials-3693170-supplementary.pdf]

Article

Supplementary materials

Influence of Temperature on the Optical Properties of Ternary Organic Thin Films for Photovoltaics

Gabriela Lewinska[1*], Jerzy Sanetra[2], Konstanty W. Marszalek[1,2,] Alexander Quandt[3,4] and Bouchta Sahraoui[3]

1 AGH University of Krakow, Institute of Electronics, 30 Mickiewicza Ave, 30-059 Krakow, Poland
2 Advanced Diagnostic Equipment sp. zoo, Włodzimierza Tetmajera 79, 31-352 Krakow, Poland
3 University of Angers, LPhiA, SFR matrix, 49045 Angers cedex 2, Bd Lavoisier 2, France
4 School of Physics, University of the Witwatersrand, Private Bag 3, Wits, 2050, South Africa
* glewinska@agh.edu.pl

Academic Editor: Firstname Lastname

Received: date
Revised: date
Accepted: date
Published: date

Citation: To be added by editorial staff during production.

Copyright: © 2025 by the authors. Submitted for possible open access publication under the terms and conditions of the Creative Commons Attribution (CC BY) license (https://creativecommons.org/licenses/by/4.0/).

Table S1. Gaussian oscillator parameters values for PTB7 Y5 PCBM

**PTB7 Y5 PCBM 30 deg heating**			
	Amplitude (a.u.)	Br Gaussian broadening (eV)	En centre energy (eV).
**1**	23.62	0.3224	1.818
**2**	-100	0.1934	0.150
**3**	-22.42	0.3112	1.822
**4**	0.1930	0.149	0.149
**5**	541.2	0.1255	0.134
**6**	1.446	12.37	3.098
**7**	-100	0.1922	0.149
**PTB7 Y5 PCBM 120 deg heating**			
	Amplitude (a.u.)	Br Gaussian broadening (eV)	En centre energy (eV).
**1**	24.22	0.3606	1.822
**2**	-100.0	0.1657	0.126
**3**	-22.05	0.3417	1.833
**4**	-100.0	0.1658	0.126
**5**	439.1	0.1142	0.140
**6**	2.010	17.8491	5.735
**7**	-100.0	0.1657	0.126
**PTB7 Y5 PCBM temp 30 deg after cooling**			
	Amplitude (a.u.)	Br	En centre energy (eV).
**1**	23.67	0.3224	1.818
**2**	-100	0.1934	0.1500
**3**	-22.42	0.3112	1.822
**4**	-100	0.1930	0.149
**5**	541.2	0.1255	0.138
**6**	1.440	12.37	3.098
**7**	-100.0	0.1922	0.149

Table S2. Gaussian oscillator parameters values for PTB7 Y6 PCBM

PTB7 Y6 PCBM 30 deg heating			
	Amplitude (a.u.)	Br Gaussian broadening (eV)	En centre energy (eV).
**1**	23.63	0.3360	1.737
**2**	-100.0	0.1499	0.112
**3**	-22.59	0.3245	1.746
**4**	-100.0	0.1499	0.1120
**5**	314.1	0.09720	0.1640
**6**	1.813	9.638	2.421
**7**	-100.0	0.1499	0.1120
**PTB7 Y6 PCBM 120 deg heating**			
	Amplitude (a.u.)	Br Gaussian broadening (eV)	En centre energy (eV).
**1**	23.71	0.2825	1.683
**2**	-99.85	0.2090	0.1530

**3**	-22.62	0.2699	1.689
**4**	-99.84	0.2090	0.153
**5**	272.9	0.0945	0.340
**6**	2.0239	11.3981	2.757
**7**	-99.85	0.2090	0.153
**PTB7 Y6 PCBM temp 30 deg after cooling**			
	Amplitude (a.u.)	Br Gaussian broadening (eV)	En centre energy (eV).
**1**	23.95	0.2387	1.638
**2**	-98.44	0.2264	0.159
**3**	-22.66	0.2246	1.643
**4**	-98.44	0.2264	0.159
**5**	276.8	0.0952	0.360
**6**	2.099	2.730	2.730
**7**	-98.44	0.2264	0.159

Table S3. Gaussian oscillator parameters values for PTB7th Y5 PCBM

**PTB7th Y5 PCBM 30 deg heating**			
	Amplitude (a.u.)	Br Gaussian broadening (eV)	En centre energy (eV).
**1**	23.54	0.3781	1.780
**2**	-100.0	0.1724	0.132
**3**	-22.55	0.3641	1.790
**4**	-100.0	0.1725	0.132
**5**	491.7	0.1121	0.128
**6**	1.616121	10.6306	3.676
**7**	-100.0	0.1723	0.132
**PTB7th Y5 PCBM 120 deg heating**			
	Amplitude (a.u.)	Br Gaussian broadening (eV)	En centre energy (eV).
**1**	23.56	0.3958	1.763
**2**	-100.0	0.1499	0.113
**3**	-22.54	0.3816	1.773
**4**	-100.0	0.1500	0.113
**5**	416.8	0.1027	0.132
**6**	10.56	10.561	3.461
**7**	-100.0	0.1500	0.113
**PTB7th Y5 PCBM temp 30 deg after cooling**			
	Amplitude (a.u.)	Br Gaussian broadening (eV)	En centre energy (eV).
**1**	23.97	0.2293	1.631
**2**	-98.34	0.2286	0.160
**3**	-22.69	0.2145	1.635
**4**	-98.34	0.2286	0.1600
**5**	277.8	0.0953	0.3610
**6**	2.113	11.12	2.731
**7**	-98.34	0.2286	0.160

Table S4. Gaussian oscillator parameters values for PTB7th Y6 PCBM

**PTB7th Y6 PCBM 30 deg heating**			
	Amplitude (a.u.)	Br Gaussian broadening (eV)	En centre energy (eV).
**1**	23.54	0.3781	1.780
**2**	-100.0	0.1724	0.1320
**3**	-22.55	0.3641	1.790
**4**	-100.0	0.1725	0.132
**5**	491.7	0.1121	0.128
**6**	1.616	10.63	3.676
**7**	-100.0	0.1723	0.132
**PTB7th Y6 PCBM 120 deg heating**			
	Amplitude (a.u.)	Br Gaussian broadening (eV)	En centre energy (eV).
**1**	23.56	0.3958	1.763
**2**	-100.0	0.1499	0.113
**3**	-22.54	0.3816	1.773
**4**	-100.0	0.1500	0.1130
**5**	416.802725	0.1027	0.132
**6**	1.601535	10.5612	3.461
**7**	-100.0	0.1500	0.113
**PTB7th Y6 PCBM temp 30 deg after cooling**			
**Amplitude (a.u.)**	Br Gaussian broadening (eV)	En centre energy (eV).	Amplitude (a.u.)
**1**	23.974425	0.2293	1.631
**2**	-98.344187	0.2286	0.160
**3**	-22.698666	0.2145	1.635
**4**	-98.344158	0.2286	0.160
**5**	277.813976	0.0953	0.361
**6**	2.112862	11.1185	2.731
**7**	-98.344170	0.2286	0.160

Dispersion models are mathematical representations of how a material's refractive index (and absorption) varies with light energy (or wavelength), such as Tauc-Lorentz or Cody-Lorentz. The dispersion curve's shape is influenced by the characteristics of these models, such as amplitude.
In these models, negative amplitudes are employed to depict complicated dispersion curve forms, while being physically contradictory (because they do not represent "negative" absorption). In order to adequately reflect the behaviors of real materials, the model may need to employ specific mathematical "tricks."
Light is reflected and refracted several times in complicated systems and thin layers. Unusual spectrum forms may result from these interferences. To fit the measured data in these circumstances, a dispersion model with negative amplitude might be required.
In thin layers and complex systems light is reflected and refracted multiple times. These interferences can lead to unusual spectrum shapes. In such situations, a dispersion model with negative amplitude may be necessary to fit the measured data.
Negative amplitude can compensate for other model parameters, such as energy shift or peak width, to achieve the best fit to the experimental data.

Table S5. Efficiency values in cells with thermal treatment

Reference	Temperature annealing	Active layer	efficiency
**Zheng et al. [32]**	177°C	pristine ClAlPc films and ClAlPc:C60 blend	3.58%
**Green et al. [33]**	120°C for 10 minutes	poly(3-hexylthiophene) and [6,6]-phenyl- C61 butyric acid methyl ester.	2.35%
**Thao et al. [34]**	60–80°C	poly(3-hexylthiophene) (P3HT) and P3HT+nc-TiO2 (PTC), active layer P3HT:PCBM and PTC:PCBM	1.6% and 2.1%
**Miao et al. [35]**	150°C	a small molecule donor and a polymer acceptor (MD/PA-type) as the active layers (BD3T:PBN-14, BD3T:PBN-15	5.06% and 9.51%

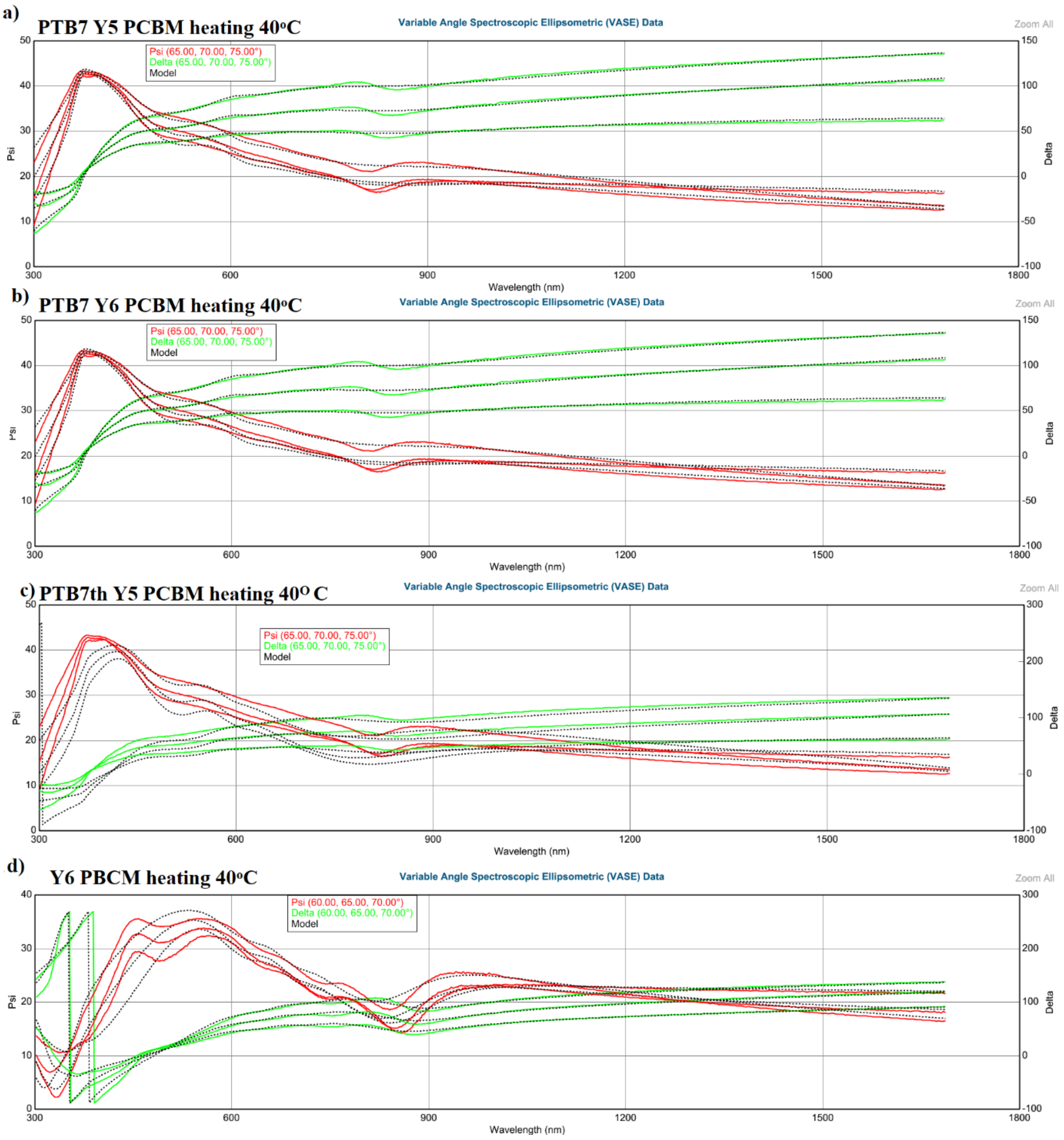

Figure S1. Ellipsometry angles with the fitted model for the considered thin films during heating for 40°C
